# The role of N-terminal phosphorylation of DGK-θ

**DOI:** 10.1016/j.jlr.2024.100506

**Published:** 2024-01-23

**Authors:** Millie X. Barbernitz, Lauren R. Devine, Robert N. Cole, Daniel M. Raben

**Affiliations:** 1 Department of Biological Chemistry, The Johns Hopkins University School of Medicine, Baltimore, MD, USA; 2 Department of Physiology and Oncology, The Johns Hopkins University School of Medicine, Baltimore, MD, USA

**Keywords:** DGK-θ, enzyme activity, membrane binding, phosphorylation, thermal stability, half-life

## Abstract

Diacylglycerol kinases (DGKs) are lipid kinases that mediate the phosphorylation of diacylglycerol (DAG) leading to the production of phosphatidic acid (PtdOH). To examine the role of phosphorylation on DGK-θ, we first identified the phosphorylated sites on endogenous DGK-θ from mouse brain and found four sites: S15, S17, which we refer to phosphomotif-1 sites, and S22 and S26 which we refer to as phosphomotif-2 sites. This study focused on the role of these phosphorylated sites on enzyme activity, membrane binding, thermal stability, and cellular half-life of DGK-θ. After generating a construct devoid of all non-catalytic phosphorylation sites (4A), we also generated other constructs to mimic phosphorylation of these residues by mutating them to glutamate (E). Our data demonstrate that an increase in membrane affinity requires the phosphorylation of all four endogenous sites as the phosphomimetic 4E but not other phosphomimietics. Furthermore, 4E also shows an increase in basal activity as well as an increase in the Syt1-induced activity compared to 4A. It is noteworthy that these phosphorylations had no effect on the thermal stability or cellular half-life of this enzyme. Interestingly, when only one phosphorylation domain (phosphomotif-1 or phosphomotif-2) contained phosphomimetics (S15E/S17E or S22E/S26E), the basal activity was also increased but membrane binding affinity was not increase. Furthermore, when only one residue in each domain mimicked an endogenous phosphorylated serine (S15E/S22E or S17E/S26E), the Syt1-induced activity as well as membrane binding affinity decreased relative to 4A. These results indicate that these endogenous phosphorylation sites contribute differentially to membrane binding and enzymatic activity.

Diacylglycerol (DAG) and phosphatidic acid (PtdOH) play important roles in a variety of signaling cascades (1, 2). Therefore, it is critical to understand the regulatory mechanisms controlling the levels of these lipids. One class of enzymes capable of coordinating the cellular levels of these two lipids is the diacylglycerol kinases (DGKs). DGKs catalyze the transfer of the γ-phosphate of ATP to the hydroxyl group of DAG which generates PtdOH (3, 4). To date, ten mammalian DGKs (α, β, γ, δ, η, κ, ε, ζ, ι, θ) have been identified, and based on their primary structures, they are classified into five types. While there is considerable data regarding the physiological roles of DGKs, our understanding of the mechanisms involved in the regulation of enzymatic activity and membrane binding is more limited. This is especially true for the lone Type V DGK, DGK-θ.

Post-translational modifications (PTMs) are well-known to play important roles in regulating enzyme activity and signaling pathways (5, 6). Among different PTMs, phosphorylation is one of the most widely observed and well-studied modifications (5, 6, 7). However, despite the popularity of PTMs, little is known about the role of PTMs on DGKs (8). Perhaps the most well-studied PTM of DGKs is phosphorylation. Seven out of ten DGKs have been shown to be phosphorylated (3, 8), and these phosphorylations were shown to modulate the enzyme activity and membrane binding. For example, mutation of sites known to be phosphorylated on DGKγ to glutamic acid led to a much higher enzymatic activity (9). We recently showed DGK-θ plays a role in modulating compensatory endocytosis in mouse central nervous system neurons (10) and is regulated by membrane lipids, such as phosphatidylserine (PtdSer) and PtdOH (11, 12) as well as the neuronal protein synaptotagmin-1 (Syt1) (13). While the role of phosphorylation on this isoform has not been thoroughly investigated, one report indicated that the phosphorylation of DGK-θ in cells leads to its association with cellular membranes (14), but the phosphorylation sites were not identified.

In this paper, we identified four endogenous phosphorylation serine residues present at the N-terminal of DGK-θ (S15, S17, S22, and S26, Fig. 1A and supplemental Fig. S1A–D) isolated from mouse brain. Given the location of these residues, we refer to S15/S17 as phosphomotif-1, and S22/S26 as phosphomotif-2 (Fig. 1A). The predicted location of these phosphomotifs in the AlphaFold structure of mouse DGK-θ is shown in Fig. 1B.

**Fig. 1:**
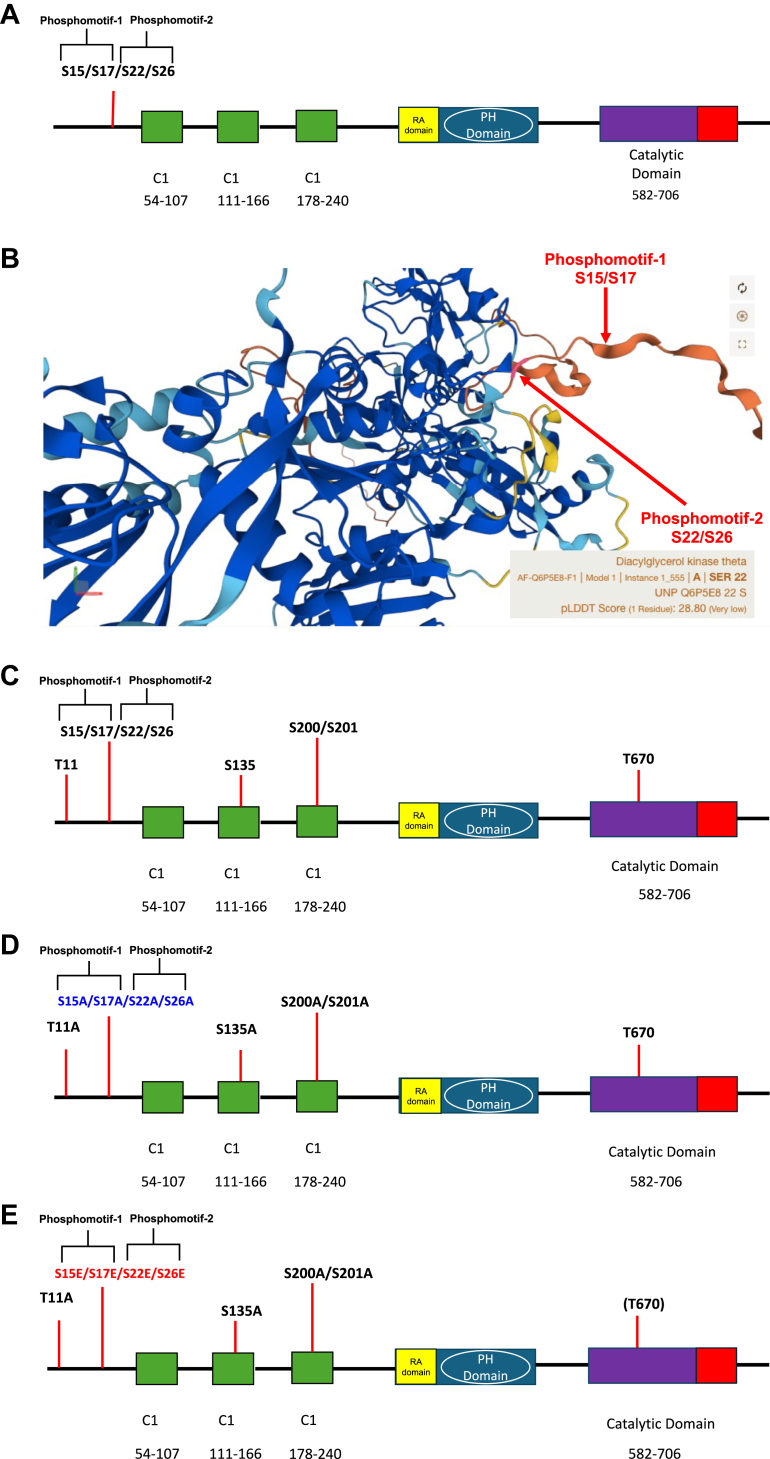
Schematics of endogenous DGK-θ phosphorylation sites and the 4A and 4E DGK-θ constructs. A: Phosphorylation sites present in endogenous DGK-θ determined by mass spectroscopy as described in the Methods. B: The predicted location of endogenous sites. C: Phosphorylated sites on overexpressed mouse DGK-θ. D: Unphosphorylated DGK-θ construct (4A) E: Phosphomimetic DGK-θ construct (4E).

To examine the role of phosphorylation in these domains on thermal stability, cellular half-life, membrane binding, and catalytic activity, we generated DGK-θ constructs that mimic unphosphorylated and phosphorylated enzymes. Our data demonstrate that an increase in membrane affinity requires the phosphorylation of all four endogenous sites as the phosphomimetic 4E, but not other phosphomimietics, has a lower K_D_ for liposomes. Further, 4E also shows an increase in basal activity as well as an increase in the Syt1-induced activity compared to 4A. It is noteworthy, that these phosphorylations had no effect on the thermal stability or cellular half-life of this enzyme. Interestingly, when only one phosphorylation motif (phosphomotif-1 or phosphomotif-2) contained phosphomimetics (S15E/S17E or S22E/S26E), the basal activity is also increased but membrane binding affinity was not increased. Furthermore, when only one residue in each domain mimicked an endogenous phosphorylated residue (S15E/S22E or S17E/S26E), the Syt1-induced activity and membrane binding affinity both decreased relative to 4A. These results indicate that the endogenous phosphorylation sites contribute differentially to membrane binding and enzymatic activity.

## Materials and Methods

### Reagents

L-α-phosphatidylcholine (PtdCho) (Brain, Porcine), L-α-phosphatidylinositol (Liver, Bovine), Ceramide (Brain, Porcine), cholesterol (ovine wool, >98%), 1-palmitoyl-2-oleoyl-sn-glycero-3-phospho-L-serine (POPtdSer, sodium salt), 1-palmitoyl-2-oleoyl-sn-glycero-3-phosphoethanolamine (POPtdEth), Sphingomyelin (SM, Brain, Porcine), 1-2-dioleoyl-sn-glycerol (DOG), 1-Oleoyl-2-[12-[(7-nitro-2-1,3-benzoxadiazol-4-yl)amino]dodecanoyl]-sn-Glycero-3-Phosphocholine (NBD-PtdCho), 1,2-dioleoyl-sn-glycero-3-phosphocholine (DOPtdCho) were purchased from Avanti Polar Lipids Inc. Aluminum-backed silica gel 60 TLC plates, methanol and B. cereus phospholipase C were purchased from Sigma-Aldrich. PEI MAX-Transfection Grade Linear Polyethylenimine Hydrochloride (MW 40,000) was purchased from Polysciences. Trypsin and Halo-TMR were purchased from Promega. The Q5 site-directed mutagenesis kit was purchased from New England Biolabs. The vector Dgkq_OMu18546C_pcDNA3.1(+)-N-DYK (*mus musculus* DGK-θ) was prepared and purchased from GenScript. All mutations were made with this vector.

### Animals

Mice brains used in this study were gifts from Dr. Nathan Archer’s lab, and wild-type female mice aged 10–12 weeks were used. All experimental procedures were reviewed and approved by the Johns Hopkins University Institutional Animal Care and Use Committee (protocol # MO21M378).

### Cell culture and transfection

HEK293T cells were grown in DMEM (Dulbecco’s modified Eagle’s medium, Thermo Fisher) containing 10% FBS (fetal bovine serum) and 1% penicillin-streptomycin. After cells reached approximately 80%–90% confluence, they were trypsinized and 9 x10^6^ cells were plated into 225 cm flasks and grown 18–24h when the cells were approximately 50%–70% confluency. Cells were then transfected with 15 μg of various DGK-θ constructs shown in Table 1 using PEI. After 3–4 days post-transfection, cells were harvested and stored at −80°C.

**Table 1:** The mutations that were used in this study

4A	T11A/S135A/S200A/S201A/**S15A,17A,22A,26A**
4E	T11A/S135A/S200A/S201A/**S15E**,**17E,22E,26E**
S15E/S17E	T11A/S135A/S200A/S201A/**S15E,17E**,22A,26A
S22E/S26E	T11A/S135A/S200A/S201A/S15A,17A,**22E,26E**
S15E/S22E	T11A/S135A/S200A/S201A/**S15E**,17A,**22E**,26A
S17E/S26E	T11A/S135A/S200A/S201A/S15A,**17E**,22A,**26E**

The bold letters indicate where the mutations were.

### Isolation of mouse brain DGK-θ

To identify the endogenous phosphorylation sites in mouse brain DGK-θ, 1–2 ml of RIPA buffer (pH 7.6, Thermo Scientific) was added to each mouse brain and homogenized using a 7 ml mortar and pestle until it appeared homogenous. It was then incubated in the RIPA buffer at 4°C for 15 min followed by 15s sonication using a Branson Digital Sonifier SFX250 equipped with half half-inch horn. The lysate was then centrifuged at 17000g for 10 min, and the enzyme was immunoprecipitated from the supernatant using an anti-DGK-θ antibody purchased from Santa Cruz. After five washes using RIPA buffer, DGK-θ along with other proteins on the beads were eluted off by boiling sample buffer at 85°C for 5 min. The immunoprecipitated sample was subjected to 8% SDS-PAGE, and the DGK-θ band was identified by Western blot analysis of a sister blot. The DGK-θ band was cut from the gel, reduced, alkylated, and trypsin digested as described below.

### Purification of overexpressed DGK-θ

HEK293T cells transfected with different FLAG-tagged mouse DGK-θ constructs (Table 1) were harvested and lysed by incubating them in lysis buffer (50 mM HEPES, 150 mM NaCl, 0.1% NP-40, pH 8) for 20 min at 4°C followed by 10s sonication using a Branson Digital Sonifier SFX250 equipped with half inch horn. The lysate was then centrifuged at 17000g for 10 min. The FLAG-tagged DGK-θ was purified from the supernatant by incubation with anti-FLAG magnetic beads for 1 h. The collected beads were washed 5 times with 1 ml of a wash buffer (20 mM HEPES pH 8, 500 mM NaCl, 1 mM EDTA, 0.5 mM EGTA, 2 mM DTT, 0.1% DDM). The FLAG-tagged DGK-θ was eluted from the beads by incubating them with free FLAG peptide for 30 min in 4°C.

### In-gel digestion

Immunoprecipitated endogenous mouse brain DGK-θ or 2 μg of purified overexpressed FLAG-tagged DGK-θ in Laemmli sample buffer (Bio-Rad catalog number 1610747, 4x sample buffer) was incubated for 3–5 min at 90^0^C after the addition of BME (beta-mercaptoethanol) and then loaded on an 8% Bolt gel (Thermo Scientific). The protein was chromatographed using NuPage MES SDS running buffer (pH 7.3). The DGK-θ band, at approximately 110 KDa (verified by a sister Western blot), was cut from the gel and reduced using 10 mM dithiothreitol (DTT) in 50% methanol for 30 min at 60°C, followed by 55 mM iodoacetamide (IAA) in dark for 15 min at room temperature (RT). 100% acetonitrile (ACN) was added for 5 min to shrink the gel. After carefully removing the liquid, 20 ng/μl trypsin (in the TEAB, pH 8 buffer) was added to the gel band on ice and then incubated overnight at 37°C. The next day, the buffer in the tube was collected and the peptides were extracted from the gel slices by combining the digestion buffer with consecutive extractions, twice with 60% ACN, 0.1% TFA (trifluoroacetic acid) for 15 min with shaking, and then twice with 100% ACN for 5 min. The extracted peptides were dried using a Speed Vac concentrator.

### Mass spectrometry of DGK-θ

DGK-θ trypitic peptides isolated above were analyzed by reverse-phase chromatography/tandem mass spectrometry on an EasyLC nano HPLC interfaced with an Orbitrap Fusion Lumos mass spectrometer (Thermo Scientific). The peptides were separated using a gradient of 0%–100% acetonitrile in 0.1% formic acid over 120 min at 300 nl/min. A 75 μm x 25 cm column (ESI Source Solutions) was packed in-house with ReproSIL-Pur-120-C18-AQ (3 μm, 120 Å bulk phase, Dr Maisch). Survey scans of precursor ions were acquired from 350-1800 m/z at 120,000 resolution at 200 m/z, automatic gain control (AGC) of 1 × 10^6^, and an RF lens setting of 50%. Precursor ions were individually isolated within 1.6 m/z by data-dependent acquisition with a 30s dynamic exclusion, within a 3 s cycle time. Precursors were fragmented using an HCD activation with a collision energy of 35. Fragment ions were analyzed at 60,000 resolution, AGC of 1 × 10^5,^ and intensity threshold of 2 × 10^4^, with a 200 ms max injection time.

Tandem mass spectra were analyzed using Mascot (Matrix Science, London, UK; version Mascot/with Mascot version 2.7.0 in Proteome Discoverer 2.4.0.305. Mascot was set up to search RefSeq2021_204_mus_musculus (92,666 entries), assuming the digestion enzyme was trypsin. Mascot was searched with a fragment ion mass tolerance of 0.0100 Da and a parent ion tolerance of 6.0 PPM. Carbamidomethyl of cysteine was specified in Mascot as a fixed modification. Deamidation of asparagine, oxidation of methionine, and phosphorylation of serine, threonine, and tyrosine were specified in Mascot as variable modifications.

Peptide identifications from the Mascot searches were processed and imported into Scaffold (Proteome Software Inc.), with peptide validation (1% false-discovery rate) and protein inference (95% confidence) by PeptideProphet (15) and ProteinProphet (16), respectively.

Precursor areas were also calculated for the phosphorylated peptides. Using Proteome Discoverer (version 2.5.0.400) with Minora feature detector, peptides were searched against UP589_M_musculus database (92,707) through mascot with target decoy validation. The enzyme was set to trypsin, with one missed cleavage allowed. Fragment mass tolerance of 0.033 Da, with a precursor mass tolerance of 5pmm allowed. Static modification of carbamidomethyl on cysteine, and variable modifications included oxidation of (m), deamidation (NQ), and Phosphorylation (ST). A high false discovery rate was set to 1.0%, and normalized and scaled precursor areas were provided for each of the unique phosphorylated peptides.

### Mutagenesis

The phosphorylation sites mapped from mass spectrometry above were mutated using a Q5 site-directed mutagenesis kit (New England Biolabs) according to the manufacturer's protocol. In brief, 1 ng of the Dgkq_OMu18546C_pcDNA3.1(+)-N-DYK template was PCR amplified. The resulting PCR product was incubated with KLD enzyme mix (a mixture of kinase, ligase, and DpnI enzymes) for 5 min at 25°C. The KLD reaction product was used for transformation. Following the transformation, 3 to 4 colonies were picked and plasmids were isolated using GeneJet miniprep kit (Thermo Scientific). Isolated plasmids were sequenced for verification in the Johns Hopkins Sequencing Facility.

### Preparation of liposomes

Lipids dissolved in chloroform were dried under nitrogen and stored under vacuum at 4° C overnight to remove residual CHCl_3_. The lipid films were rehydrated in DGK-75 assay buffer (50 mM HEPES, 150 mM NaCl, pH 8.0) for 30 min at 37°C. During rehydration, samples were vortexed and sonicated (30 s) every 10 min to completely disperse the lipid pellet. The resulting vesicles were extruded at 37°C–40°C through a 0.1 μm polycarbonate membrane using an Avanti mini extruder and used the same day. Two liposomes were used in our studies. Control liposomes containing only DOPtdCho were used for surface plasmon resonance (SPR) only, and brain liposomes were used for SPR and enzymatic studies. The lipid composition of the brain liposomes mimicked the lipid composition found in brain synaptosomes (17) and consisted of: brain PtdCho: POPtdEth: POPtdSer: cholesterol: ceramide: brain SM: PtdIns: DOG (24.5:24: 9.6: 21.5: 6: 4.3: 2.1:8, mol%).

### DGK-θ kinase assay

The DGK-θ kinase assay was performed using a recently developed NBD-based fluorescence assay (18). Briefly, NBD-DAG was produced by phospholipase C-mediated hydrolysis of NBD-PtdCho as described (18). Purified DGK-θ was incubated with 3 mM brain liposomes supplemented with NBD-DAG (at 6% of the total DAG), 1 mM ATP, and 1.5 mM MgCl_2_, with or without 0.02 μg/μl Syt-1 (total reaction volume was 50 μl) at 37°C for 20 min. The reactions were stopped by adding 100 μl of methanol followed by a Bligh-Dyer extraction of total lipids. The organic phase was dried under nitrogen. To separate the NBD-PtdOH from the NBD-DAG, the dried lipids were solubilized in chloroform:methanol (2:1 v/v), spotted on a silica gel 60 TLC plate, and developed using chloroform: methanol: 80% acetic acid (65:15:5 v/v). The NBD-PtdOH generated in the kinase reaction was scanned by Typhoon RGB (Cytiva Life Sciences) and quantified by ImageJ software. In all assays, a standard curve of NBD-PtdOH was used for quantification. A blank region of the TLC plate was quantified and used as a background which was subtracted from all samples.

### SPR binding assay

The dissociation constant (K_D_) for membrane binding was assessed kinetically by SPR using a Biacore 8K instrument. All experiments were performed at 37°C. For these studies, control and brain liposomes described above were used as the ligands and purified FLAG-tagged DGK-θ constructs were used as the analyte. Liposomes were immobilized on an L1 sensor chip (Cytiva Life Sciences). The sensor chip surface was rinsed with two injections of 20 mM CHAPS before injecting the liposomes. 3 mM liposomes in DGK-75 buffer were then injected over the L1 chip for 60 at a 10 μl/min flow rate. Typical RU values for control DOPtdCho and brain liposomes were approximately 8000 resonance units (RU) which is similar to lipid immobilization values as previously reported (19, 20). DGK-75 buffer was used as a running buffer.

To examine the association and dissociation rate constants, various DGK-θ constructs flowed over the liposome-immobilized chip in DGK-binding buffer (25 mM Tris, 0.001% Triton, 0.0005% DDM, pH 8.0). Before injecting a DGK-θ construct, the chip surface was rinsed with the running buffer until the baseline was stable. Various concentrations of a purified DGK-θ construct were then flowed over the chip separately at a flow rate of 30 μl/min, for 120s to assess binding rate constants. The DGK-θ constructs were allowed to dissociate for 300s to assess dissociation rate constants. To regenerate the L1 sensor chip, the chip was washed with 20 mM CHAPS (10 μl/min, 60s). The association rate constant (k_on_) and the dissociation rate constant (k_off_) were examined through a kinetic fitting mode using Insight Evaluation Software (Cytiva Life Sciences).

### Thermal stability assay

Differential scanning fluorimetry (DSF) (21) was used to evaluate the thermal stability of DGK-θ constructs. In brief, 20 μl reactions in white 96 well unskirted PCR plates (Thermo Scientific) by mixing 1.5 μg of a FLAG-tagged DGK-θ in the DGK-75 buffer containing 0.005% DDM, pH 8.0. 2 μl of 50X SYPRO orange dye (Invitrogen) was then added. Thermal scanning was performed from 25°C to 99°C at a 1°C/min temperature gradient, using a CFX9 Connect Real-Time PCR Detection System (Bio-Rad). The melting curves were constructed from the fluorescence intensity versus temperature and the protein melting temperature (T_m_) was calculated using GraphPad Prism 10.2.0 (GraphPad Software, USA).

### Half-life determination

To assess the cellular half-life of the DGKs, Halo TMR labeling (Promega) was used as previously described by Merrill *et al.* (22). Briefly, HEK293T cells were transfected with Halo tagged mouse DGK-θ constructs where the Halo Tag was placed at the N-terminus before the FLAG tag. 48–72h post-transfection, cells were incubated with 50 nM of tetramethylrhodamine (TMR) ligand at 37°C for 30 min. The media was then removed, and cells were washed twice with PBS and 10 μM 7-bromo 1-heptanol (7BRO) for up to 48h. At various times, the cells were harvested in PBS buffer followed by centrifugation at 1000g for 5 min and lysed in RIPA buffer. Following lysis, equivalent amounts of cell lysate in Laemmli sample buffer were loaded onto an 8% Bolt gel and subjected to SDS-PAGE as described earlier. The DGK-θ-TMR fluorescence was quantified by scanning the gel on a Typhoon GRB followed by ImageJ quantification. Total protein concentrations loaded on the gels were then quantified using Coomassie gel staining and quantified by ImageJ software.

## Results

### Identification of mouse brain DGK-θ phosphorylation sites

To direct our studies, we identified the DGK-θ phosphorylation sites present endogenously in mouse brains as described in the Method section. Four endogenous phosphorylation sites were identified: S15, S17, S22 and S26 (Fig. 1A and supplemental Fig. S1A–D) and we refer to them as phosphomotif-1 (S15/S17) and phosphomotif-2 (S22/S26). All mass spectrometry data was repeated at least two times with essentially the same results. A qualitative assessment of the phosphorylation sites by precursor area analysis showed that the phosphorylation of phosphomotif-1 is approximately 10% while the phosphorylation of phosphomotif-2 is approximately 2% compared with non-phosphorylated peptides. We note, the caveat with this approach is that the ionization efficiency of the phosphorylated forms is likely to be lower than the non-phosphorylated forms, suggesting this might be a low estimate of the actual endogenous phosphorylation levels.

### Generation of a unphosphorylated enzyme and phosphomimetics

The major goal of this study was to examine the role of DGK-θ phosphorylations in regulating its protein stability, cellular half-life, membrane binding, and catalytic activity, Our strategy was to generate DGK-θ constructs that lacked phosphorylation in both phosphomotifs 1 and 2 (4A) as well as DGK-θ constructs that mimicked the phosphorylations found in these domains. To accomplish this, we used DGK-θ constructs that were overexpressed in HEK293T cells. It was therefore important to identify the phosphorylation pattern in overexpressed DGK-θ. We discovered five additional sites not found in the endogenous enzyme (T11, S135, S200/201, and T670, Fig. 1B). To mimic the unphosphorylated endogenous enzyme (4A), all residues phosphorylated in the overexpressed enzyme (Fig. 1C), except T670, were mutated to alanines (Fig. 1D). To make phosphomimetics (23), the endogenous sites in 4A were mutated to glutamate (Fig. 1E and Table 1). We note that T670 was not mutated as mutation of this residue resulted in the complete loss of DGK-θ activity (data not shown).

### Phosphorylation had no effect on the thermal stability of DGK-θ

**Fig. 2:**
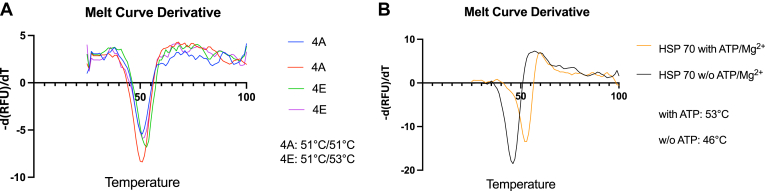
Phosphorylations do not appear to affect the DGK-θ′s thermal stability. Differential scanning fluorimetry (DSF) was used to evaluate the thermal stability of 4A and 4E constructs as described in Materials and Methods using SYPRO orange dye (Invitrogen). Briefly, thermal scanning was performed from 25°C to 99°C (1°C/min temperature gradient) using a CFX9 connect real-time polymerase chain reaction instrument (Bio-Rad). The melting curves were constructed from the fluorescence intensity versus temperature and the protein melting temperature ‘T_m_’ was calculated using a prism. A: 4A and 4E melting temperature. This was repeated at least three times with essentially the same results. B: The melting temperature for HSP-70 with and without ATP/Mg^2+^ (100 μM ATP/500 μM MgCl_2_) added in the buffer.

Phosphorylation has been known to affect the thermal stability of some proteins (24, 25). Huang *et al.* found that among 2883 phosphorylation sites on proteins expressed in HEK293T cells, 719 phosphorylations appear to have a significant influence on the thermal stability of proteins (26, 27). We therefore examined the role of phosphorylation on the thermal stability of DGK-θ using differential scanning fluorimetry (21). The data showed that unphosphorylated 4A and the phosphomimetic 4E, have very similar melting temperatures of 51 °C and 52°C, respectively (Fig. 2A). This is similar to the reported thermal shift temperature for most proteins (27). As a positive control, we examine the melting temperature of HSP70 in the presence and absence of ATP/Mg^2+^ (100 μM ATP/500 μM MgCl_2_). As shown in Fig. 2B, the addition of ATP/Mg^2+^ shifts the melting temperature of HSP70 by 7°C from 46 °C to 53 °C. These data indicate that the endogenous phosphorylation sites on DGK-θ do not significantly influence its thermal stability (Fig. 2A).

### Phosphorylation did not influence the cellular half-life of DGK-θ

**Fig. 3:**
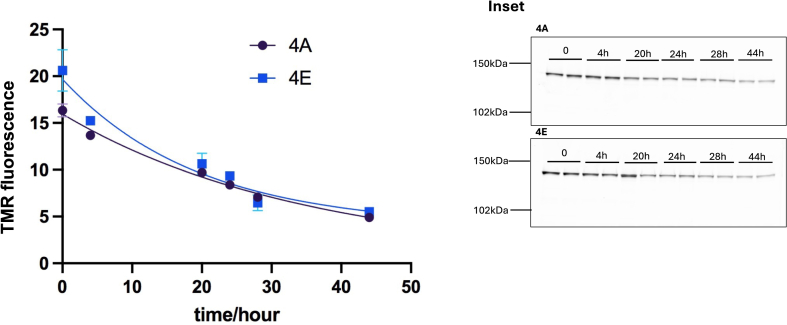
Phosphorylations do not influence the cellular half-life of DGK-θ. HEK293T cells were transfected with Halo-tagged mouse DGK-θ constructs using PEI. 48–72 h post-transfection, cells were incubated with the TMR ligand at 37°C for 30 min. Then the media was removed, and cells were washed twice with PBS and 10 μM 7-bromoheptanol (7BRO) for up to 48 h. At various times, the cells were harvested by PBS buffer followed by centrifugation at 1000g for 5 min, and then lysed in RIPA buffer. Following lysis, equivalent amounts of cell lysate were loaded onto 8% SDS-PAGE. The DGK-θ-TMR fluorescence in the gel was scanned using a Typhoon GRB (inset). The fluorescence was quantified by image J. This was repeated twice for 4A and three times for 4E with essentially the same results. Error bars indicate standard deviation.

Phosphorylation has been shown to influence the cellular half-life of some proteins (28, 29). To examine the role of phosphorylation on the cellular half-life time of DGK-θ, we used a Halo TMR labeling approach as described in Methods. In these studies, we examined the half-life time of our 4A and 4E constructs. As a control, and to determine the effectiveness of the bromo 1-heptanol (7BRO) concentration used in our experiments to block the TMR Halo signal, cells were incubated with 50 nM TMR ligand and 10 μM 7BRO simultaneously at 37°C for 30 min. Under these conditions, no fluorescence was detected (data not shown). As shown in Fig. 3, when the cellular half-life time was examined as described in Methods, there was no significant difference between 4A and 4E.

### Effect of DGK-θ phosphorylation in phosphomotifs 1 and 2 on membrane binding

There are numerous reports that demonstrate the influence of protein phosphorylation on membrane association (30, 31) including DGK-α (32, 33). With respect to DGK-θ, there is one report that correlated its translocation to the plasma membrane of A431 cells with its phosphorylation, potentially by PKC η and/or ε (14). In view of this, we examined the role of the endogenous phosphorylation sites on DGK-θ membrane binding. The association and dissociation kinetics of DGK-θ constructs binding to liposomes were quantified by SPR. We used liposomes that contained only PtdCho (control) or liposomes that mimic the lipid membrane composition of brain synaptosomes (brain liposomes) (17). As described in Methods, the DGK-θ constructs were used as analytes and their binding constants to the liposomes, used as ligands anchored to an L1 chip, were quantified.

**Fig. 4:**
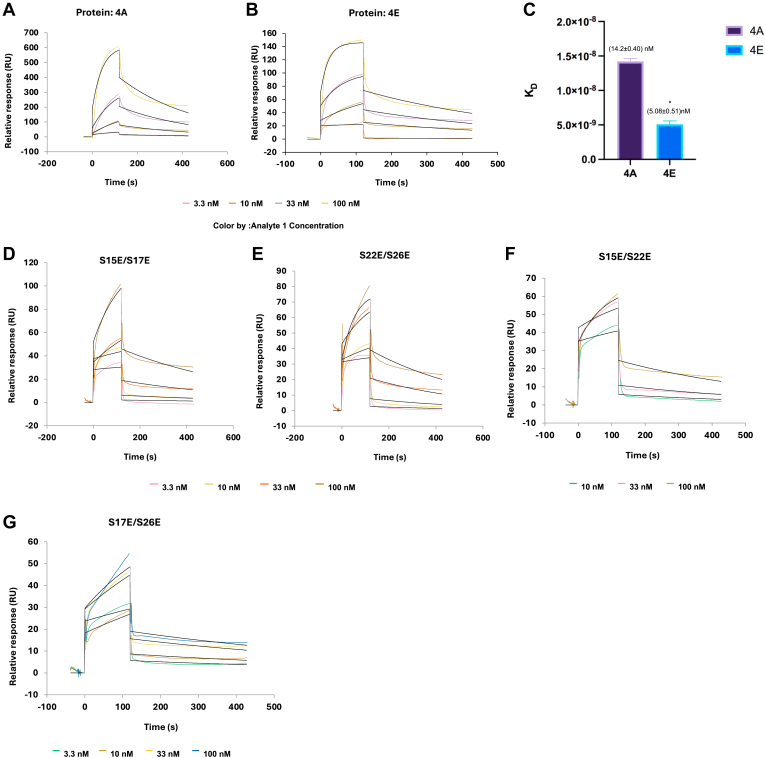
4E has the lowest K_D_ (highest affinity) for membranes. The binding of 4A and 4E to liposomes was assessed by SPR as described in Materials and Methods. A: Biosensor response data for 4A at various concentrations (from 3.3 nM to 100 nM). B: Biosensor response data for 4E at various concentrations (from 1 nM to 100 nM). C: Bar graph of K_Ds_ for 4A and 4E. Error bars indicate standard deviation. Star indicates statistical significance derived from a *t* test analysis. D–G: Biosensor response data for S15E/S17E, S22E/S26E, S15E/S22E and S17E/S26E respectively at various concentrations. The colored lines depict the actual biosensor binding data. The calculated kinetic curve fits are shown in black. These data were repeated at least twice.

The SPR curves for 4A and 4E are shown in Fig. 4A, B, respectively. The analyses of their binding kinetics showed a K_D_ of (14.2 ± 0.40) nM for 4A, while 4E bound tighter with a K_D_ of (5.08 ± 0.51) nM (Fig. 4C). To investigate which phosphorylation site(s) are responsible for this binding change, we examined the phosphomimetics shown in Table 1. As is indicated in Table 2, all four double phosphomimetics (S15E/S17E, S22E/S26E, S15E/S22E, S17E/S26E) resulted in a much higher K_D_ compared to 4E (see Fig. 4D–G for representative curves). These data suggest that an increase in binding affinity requires the phosphorylation of all four endogenous sites (4E).

**Table 2:** Membrane binding parameters for DGK-θ and mutants determined from SPR analysis

Construct	ka (M-1 S-1)	kd (S^−1^)	K_D_(M)
4A	(2.04 ± 0.02) E+05	(2.89 ± 0.11) E−3	(1.42 ± 0.04) E−8
4E	(3.78 ± 0.89) E+05	(1.9 ± 0.26) E−3	(5.08 ± 0.51) E−9
S15E/S17E	(5.76 ± 0.78) E+04	(1.69 ± 0.18) E−3	(2.95 ± 0.08) E−8
S22E/S26E	(1.5 ± 0.12) E+05	(2.89 ± 1.0) E−3	(1.97 ± 0.8) E−8
S15E/S22E	(1.14 ± 0.13) E+05	(3.02 ± 1.33) E−3	(2.61 ± 0.87) E−8
S17E/S26E	(1.45 ± 0.55) E+05	(4.92 ± 1.62) E−3	(3.43 ± 0.18) E−8

Values represent the mean ±S.D. from at least two determinations. All measurements were performed in 25 mM Tris, 0.001% Triton, 0.0005% DDM, pH 8.0.

### Effect of phosphorylation in phosphomotifs 1 and 2 on enzyme activity

Phosphorylation is well-known to modulate a number of enzymatic activities (30, 34). It is not surprising, therefore, that there are several reports demonstrating an effect of phosphorylation on the activity of DGKs. Phosphorylation of Y335 in DGK-α mediates its enzymatic activation and membrane biding (35). Mutation of S776 and S779 to glutamate (phosphomimetic) of DGK-γ resulted in a higher activity compared to an alanine mutant (9).

**Fig. 5:**
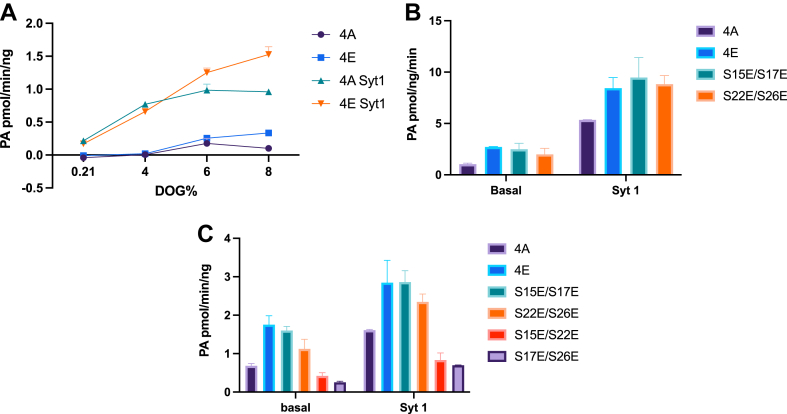
Basal and Syt1-induced enzyme activities. Enzymatic activities of DGK-θ constructs were examined using our NBD-DAG kinase assay as described in Methods. A: Purified 4A or 4E was incubated with different DAG concentrations (2 mol% to 8 mol%) in brain liposomes. These data were repeated four times with essentially the same results. Error bars indicate standard deviation. B and C: Basal and Syt1 (0.02 μg/μl)-induced activities of 4A, 4E or different double mutations were analyzed using 8 mol% DAG in brain liposomes. These data were repeated three times with essentially the same results. Error bars indicate standard deviation.

Previous studies from our lab showed DGK-θ is activated by peptides or proteins with exposed polybasic regions such as poly-L-lysine (36). Further studies identified the endogenous activator in mammalian neurons is synaptotagmin-1 (Syt1) (13). To examine the effect of phosphorylation on the basal and Syt1-induced enzyme activity, we used the NBD-DAG-based DGK assay developed in our laboratory ((18)and see Methods). To begin, the activities of 4A and 4E were assessed at various DAG concentrations (2%–8%) (Fig. 5A). The data show that 4E had a slight increase in basal activity compared to 4A as well as a resulting increase in Syt1-indued activity at 6 mol% or higher.

To further examine the roles of DGK-θ phosphorylation on its enzymatic activity, we assessed the effect of various phosphomimetics (Table 1) on basal and Syt1-induced activities. As is shown in Fig. 5B, 4E and phosphomimetics in both phosphomotif-1 residues (S15E/S22E), and phosphomotif-2 residues (S22E/S26E) resulted in increased basal leading to an increase in the Syt1-stimulated activities compared to 4A. Interestingly, a phosphomimetic of one residue in each phosphomotif combined (S15E/S22E and S17E/S26E) did not show an increase, and in fact often a decrease, in basal and resulting Syt1-stimulated activities (Fig. 5C).

We again note that T670 was not mutated in this study. This is because this residue is in the catalytic domain of this enzyme, and mutation of this residue to alanine resulted in complete loss of catalytic activity. The reason for this is unclear as it was not found to be phosphorylated in the endogenous enzyme (Fig. 1A). It may be due to a low stoichiometry of phosphorylation in the endogenous enzyme.

## Discussion

DGK is a lipid kinase that transfers γ-phosphate from ATP to diacylglycerol (DAG) leading to the generation of PtdOH. Presently, there is limited information regarding the role of post-translational modifications in regulating DGKs. DGK-θ, the lone member of the type V DGKs, was reported to be phosphorylated and this phosphorylation facilitated its membrane translocation (14). Aside from these data, our understanding of the phosphorylation sites and their function(s) remains unknown.

In this study, we identified four endogenous phosphorylation sites on DGK-θ present in the mouse brain (S15, S17, S22, and S26) which we define as phosphomotif 1 (S15 and S17) phosphomotif 2 (S22 and S26). Interestingly, we found five potential additional phosphorylation sites (T11, S135, S200/S201, T670) on DGK-θ overexpressed in HEK 293T cells (Fig. 1). our study aimed to investigate the role of the endogenous phosphorylation sites in regulating DGK-θ′s membrane binding, catalytic activity, protein stability and cellular half-life. To do this, we used a construct of DGK-θ that lacked all phosphorylation sites (4A). We also used constructs with phosphomimetic glutamates at sites of endogenous phosphorylations, such as 4E which contained glutamates at all four endogenous phosphorylation sites. We used these constructs to assess the role of phosphorylations on thermal stability, cellular half-life, membrane binding, and enzyme activity.

Phosphorylation plays a role in the thermal stability (24, 25) and cellular half-life (28, 29) of various proteins. Our data, using differential scanning fluorimetry to assess thermal stability, and a HaloTag assay to examine cellular half-life, we found that phosphorylation of DGK-θ affects neither its thermal stability (Fig. 2) nor its cellular half-life (Fig. 3). These data suggest that phosphorylation of DGK-θ does not play a role in these processes. These data are consistent with the findings of Huang *et al.* who showed that only about 25% of the phosphorylations have a significant effect on the thermal stability out of 2883 proteins (27), and Wu *et al.* used a proteomic method, DeltaSILAC, to show that the influence of phosphosites on protein turnover is heterogeneous (37).

While phosphorylation did not affect thermal stability or cellular turnover, it did affect membrane binding and enzymatic activity. For membrane binding, using SPR analyses, the data indicated that the phosphorylation of all residues in both phosphomotifs 1 and 2 resulted in a higher affinity of membrane binding. The K_D_ of 4A was found to be 14.2 nM while K_D_ of 4E was 5.08 nM (Fig. 4A–C). Interestingly, none of the phosphomimetic 2Es increases the affinity for membrane binding (Fig. 4D-G).

Since DGK-θ is an interfacial enzyme that catalyzes the phosphorylation of DAG at a membrane interface, and phosphorylation affected the enzyme affinity for membranes (above), it was important to assess the role of the phosphorylations on basal and stimulated enzyme activity. Using our newly developed NBD enzyme activity assay (18), we found that the phosphomimetic 4E displayed an increase in both basal and resulting Syt1-induced activity compared to the non-phosphomimetic 4A (Fig. 5). This was most noticeable when DAG concentrations were 6 mol% or higher. This is interesting as this is within the range of its cellular DAG concentrations (38). It is noteworthy, that when both serines in either phosphomotif-1 or phosphomotif-2 were mutated to mimic phosphorylations (S15E/S17E, or S22E/S26E respectively) the basal catalytic activity increased even though, as noted above, membrane binding affinity was not increased (Fig. 5B). In contrast, when two serines are located in different phosphomotifs (S15E/S22E, or S27E/S26E), both basal and Syt1 induced activity decreased compared to 4A (Fig. 5C). These results indicate that the endogenous phosphorylation sites contribute differentially to membrane binding and enzymatic activity and suggest that an increase in enzymatic activity requires the phosphorylation of two serines in the same phosphomotif. It also suggests that the enzyme activity change caused by phosphorylation is not dependent on membrane binding.

It is important to note again, that T670 was the only site found to be phosphorylated in the overexpressed enzyme that was not mutated to alanine. Given that T670 resides within the putative catalytic domain, it is not surprising that mutation of this residue led to a loss in enzyme activity (data not shown). The reason for this is unclear but may suggest that this threonine is involved in a catalytic ping-pong mechanism where the γ-phosphate of ATP is transiently transferred to T670, followed by its transfer to the hydroxyl group of DAG leading to the formation of PtdOH. Future studies will resolve this question.

## Data availability

The datasets described herein are available upon request from the corresponding author.

## Supplemental data

This article contains supplemental data.

## Conflict of interest

The authors declare that they have no known competing financial interests or personal relationships that could have appeared to influence the work reported in this paper.
