# Supplementary material for: The role of N-terminal phosphorylation of DGK-θ
Source: J Lipid Res. 2024 Jan 23;65(3):100506. doi: 10.1016/j.jlr.2024.100506 (PMC10914586; doi:10.1016/j.jlr.2024.100506)
Supplement: Supplementary file 1 — Figure legend for supplemental figures [file mmc1.docx]

**Figure legend for supplemental figures**

Fig. S1. MS analysis of endogenous phosphorylation sites from mice brains. Endogenous phosphorylation sites were mapped as described in Methods section. Site specific phosphorylation was confirmed by the presence of the required fragment ions as displayed in the fragment ion tables.  A, Representative MS/MS spectrum of the phosphorylated peptide for S15. B, Representative MS/MS spectrum of the phosphorylated peptide for S17. C, Representative MS/MS spectrum of the phosphorylated peptide for S22. D, Representative MS/MS spectrum of the phosphorylated peptide for S26.
