# Supplementary material for: The role of N-terminal phosphorylation of DGK-θ
Source: J Lipid Res. 2024 Jan 23;65(3):100506. doi: 10.1016/j.jlr.2024.100506 (PMC10914586; doi:10.1016/j.jlr.2024.100506)
Supplement: S1A.pdf [file mmc2.pdf]

Figure S1A

S15

Sequence: TWPGSGSPR

S5-Phospho (79.96633 Da)

#1	b^+	b^+	Seq.	y^+	$y^+\Delta$ppm	y-Phos	y-Phos Δppm	$y\text{-}H_2O^+$	$y\text{-}H_2O^+$ Δppm	#2
1		102.05496	T							9
2	+2.31	288.13427	W	923.37711						8
3		385.18703	P	737.29779	+4.58	639.32090	+6.16	719.28723	+5.52	7
4		442.20850	G	640.24503	+4.06	542.26814	+5.64	622.23447	+6.52	6
5		609.20685	S-Phospho	583.22357	+3.72	485.24667	+6.60			5
6		666.22832	G	416.22521				398.21464	+7.01	4
7		753.26035	S	359.20374						3
8		850.31311	P	272.17172	+2.26					2
9			R	175.11895	+1.53					1

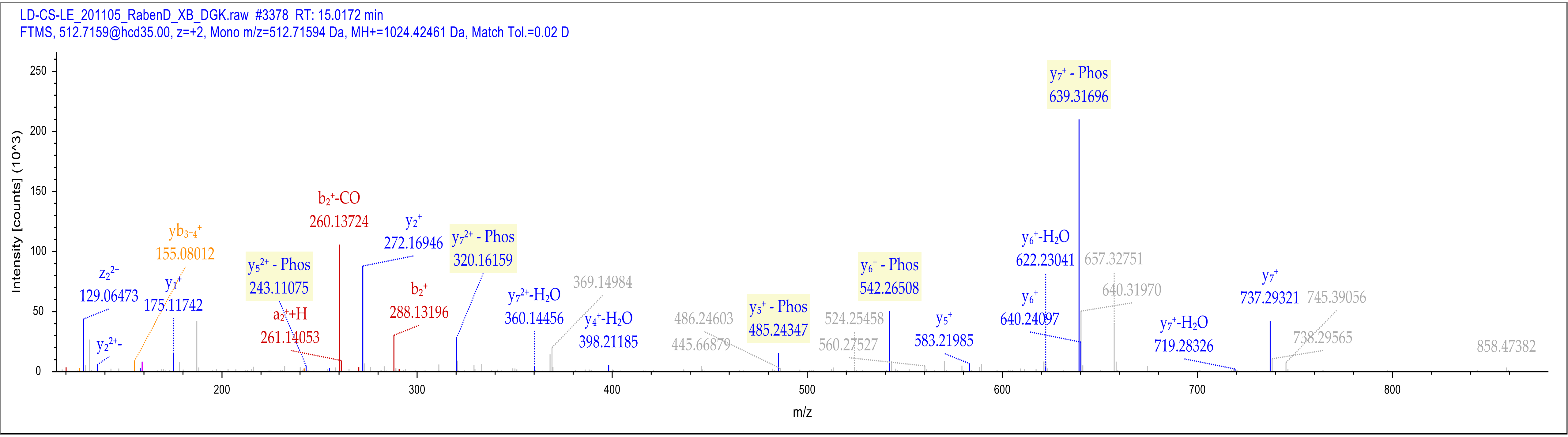

Sequence: TWPGSGSPR, S5-Phospho (79.96633 Da)

Charge: +2, Monoisotopic m/z: 512.71594 Da (-0.09 mmu/-0.17 ppm), MH+: 1024.42461 Da, RT: 15.0172 min,

Identified with: Mascot (v1.36); Ions Score:19, Ions matched by search engine: 5/64

Fragment match tolerance used for search: 0.03 Da
