# Supplementary material for: The role of N-terminal phosphorylation of DGK-θ
Source: J Lipid Res. 2024 Jan 23;65(3):100506. doi: 10.1016/j.jlr.2024.100506 (PMC10914586; doi:10.1016/j.jlr.2024.100506)
Supplement: S1B.pdf [file mmc3.pdf]

Figure S1B

S17
Sequence: TWPGSGSPR
S7-Phospho (79.96633 Da)

#1	b^+Δppm	b^+	Seq.	y^+	y^+Δppm	y-Phos	y-Phos Δppm	#2
1		102.05496	T					9
2	-1.64	288.13427	W	923.37711		825.40021		8
3		385.18703	P	737.29779	-2.72	639.32090	-3.10	7
4		442.20850	G	640.24503	-3.00	542.26814	-3.14	6
5		529.24052	S	583.22357	-2.73	485.24667	-3.46	5
6		586.26199	G	496.19154	-2.56	398.21464	-2.10	4
7		753.26035	S-Phospho	439.17008		341.19318		3
8		850.31311	P	272.17172	-1.48			2
9			R	175.11895	-1.19			1

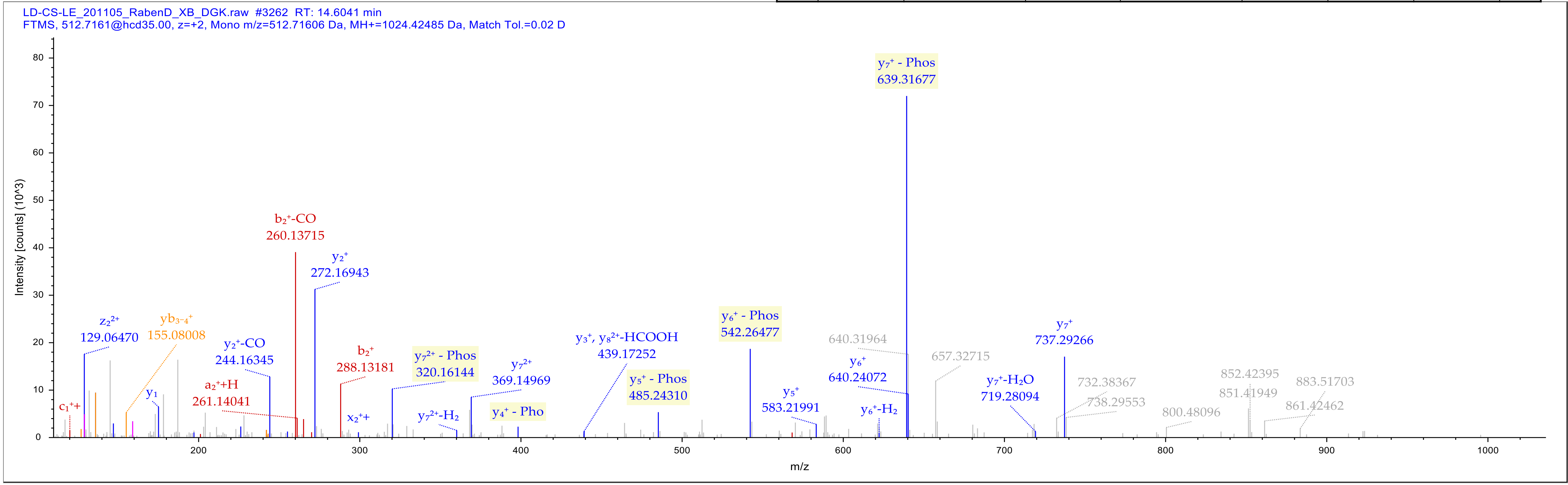

Sequence: TWPGSGSPR, S7-Phospho (79.96633 Da)
Charge: +2, Monoisotopic m/z: 512.71606 Da (+0.03 mmu/+0.06 ppm), MH+: 1024.42485 Da, RT: 14.6041 min,
Identified with: Mascot (v1.36); Ions Score:15, Ions matched by search engine: 6/64
Fragment match tolerance used for search: 0.03 Da
Fragments used for search: -H_2O; y; -NH_3; y; a; a; -H_2O; b; b; -H_2O; y
