# Supplementary material for: The role of N-terminal phosphorylation of DGK-θ
Source: J Lipid Res. 2024 Jan 23;65(3):100506. doi: 10.1016/j.jlr.2024.100506 (PMC10914586; doi:10.1016/j.jlr.2024.100506)
Supplement: S1C.pdf [file mmc4.pdf]

Figure S1C

S22

Sequence: LGSPAGSPVLGISGR

S3-Phospho (79.96633 Da)

#1	b⁺Phos Δppm	b-Phos	b- Δppm	b⁺	Seq.	y⁺	y⁺Δppm	y²⁺-Phos	y²⁺-Phos Δppm	#2
1				114.09134	L					15
2				171.11280	G	1334.64641		618.83839	-5.29	14
3		240.13427	-3.92	338.11116	S-Phospho	1277.62494		590.32766	-3.57	13
4		337.18703		435.16393	P	1110.62658	-3.96			12
5	-3.22	408.22415		506.20104	A	1013.57382	-3.58			11
6		465.24561		563.22250	G	942.53671	-2.89			10
7	-5.33	552.27764		650.25453	S	885.51524	-2.29			9
8		649.33040		747.30730	P	798.48321	-3.51			8
9		748.39882		846.37571	V	701.43045	-2.82			7
10		861.48288		959.45977	L	602.36204	-2.68			6
11		918.50434		1016.48124	G	489.27797	-3.78			5
12		1031.58841		1129.56530	I	432.25651				4
13		1118.62044		1216.59733	S	319.17244	-0.33			3
14		1175.64190		1273.61879	G	232.14042				2
15					R	175.11895				1

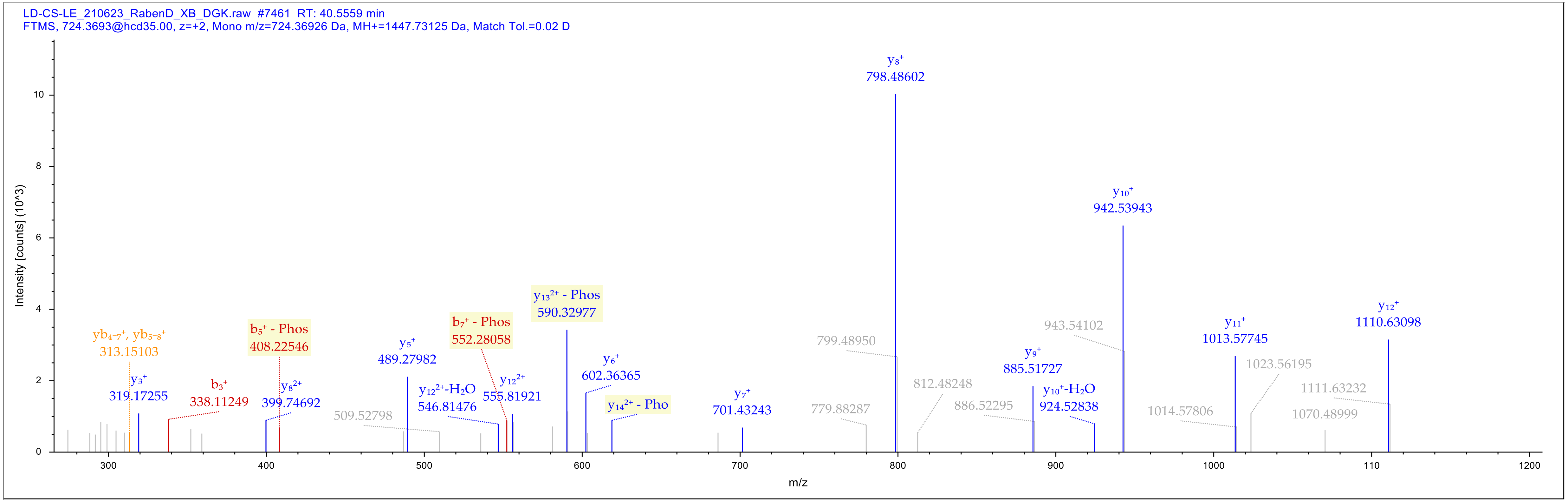

Sequence: LGSPAGSPVLGISGR, S3-Phospho (79.96633 Da)

Charge: +2, Monoisotopic m/z: 724.36926 Da (+0.39 mmu/+0.54 ppm), MH+: 1447.73125 Da, RT: 40.5559 min,

Identified with: Mascot (v1.36); Ions Score:48, Ions matched by search engine: 9/112
