# Supplementary material for: The role of N-terminal phosphorylation of DGK-θ
Source: J Lipid Res. 2024 Jan 23;65(3):100506. doi: 10.1016/j.jlr.2024.100506 (PMC10914586; doi:10.1016/j.jlr.2024.100506)
Supplement: S1D.pdf [file mmc5.pdf]

Figure S1D

S26

Sequence: LGSPAGSPVLGISGR
S7-Phospho (79.96633 Da)

#1	b-PhosΔppm	b-Phos	b⁺Δppm	b⁺	Seq.	y⁺	y⁺Δppm	y⁺-Phos	y⁺-PhosΔppm	#2
1				114.09134	L					15
2			-0.85	171.11280	G	1334.64641		1236.66951	-2.11	14
3			-1.07	258.14483	S	1277.62494		1179.64805		13
4				355.19760	P	1190.59291	-1.63	1092.61602	-1.74	12
5			-2.23	426.23471	A	1093.54015	-1.79	995.56326	-2.62	11
6				483.25617	G	1022.50304	-2.34	924.52614	-2.82	10
7	-3.77	552.27764		650.25453	S-Phospho	965.48157	-2.59	867.50468	-3.33	9
8		649.33040		747.30730	P	798.48321	-2.99			8
9	-3.16	748.39882		846.37571	V	701.43045	-3.69			7
10	-2.59	861.48288		959.45977	L	602.36204	-3.59			6
11		918.50434		1016.48124	G	489.27797	-3.08			5
12		1031.58841		1129.56530	I	432.25651	-1.39			4
13		1118.62044		1216.59733	S	319.17244	-1.46			3
14		1175.64190		1273.61879	G	232.14042	-1.22			2
15					R	175.11895	-0.90			1

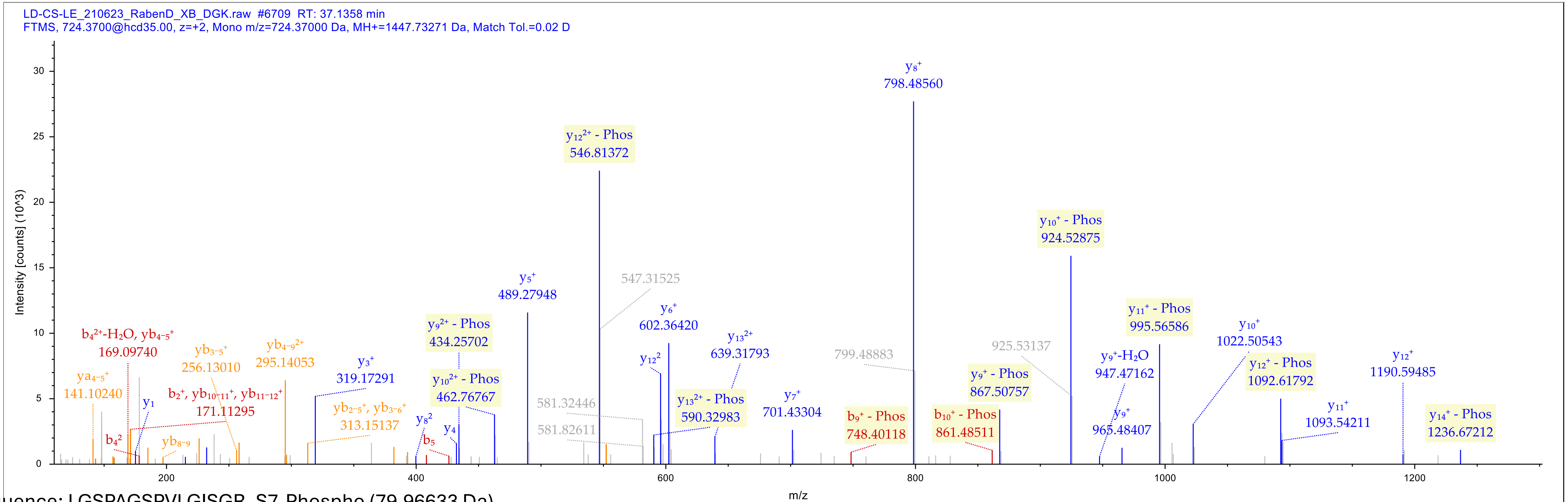

Sequence: LGSPAGSPVLGISGR, S7-Phospho (79.96633 Da)
Charge: +2, Monoisotopic m/z: 724.37000 Da (+1.12 mmu/+1.55 ppm), MH+: 1447.73271 Da, RT: 37.1358 min,
Identified with: Mascot (v1.36); Ions Score:66, Ions matched by search engine: 8/112
Fragment match tolerance used for search: 0.03 Da
